# Welcome to volume 7 of *Future Science OA*

**DOI:** 10.2144/fsoa-2020-0180

**Published:** 2020-11-09

**Authors:** Francesca Lake

**Affiliations:** 1 Future Science Group, Unitec House, 2 Albert Place, London N3 1QB, UK

Welcome to volume 7 of *Future Science OA*. While it has been an unusual year for us all, this Foreword is your usual roundup of the last year of publishing in *Future Science OA*, as well as a brief look forward to 2021.

The year 2020 saw us receive the most submissions ever – at the time of writing in mid-October, we had already received more than throughout the entirety of 2019. This increase in submissions is mirrored by an increase in publications year-on-year, and an increase in citations, to over 1000 thus far in 2020 (Dimensions data). In terms of rankings, our 2019 CiteScore is 3.1, and on track to increase for 2020.

## Content highlights

In terms of content highlights, it should be no surprise that our publications related to the COVID-19 pandemic make our most-read list for 2020. These include some thought-provoking editorials, discussing topics such as whether cannabinoids could play a role in quelling the cytokine storm [1], and whether Toll-like receptors could be prime targets to treat the disease [2].

It has been noteworthy this year that we have received a large number of submissions related to COVID-19, not all of them good – on average, across our publishing house, we have rejected 50% of these either at the desk, or post-peer review. This was not a trend specific to us – most publishers with relevant journals have seen an influx of COVID-19-relevant submissions, and the race to publish has resulted in many being of insufficient quality and rejected; notably, some have even been published and then retracted once errors have been spotted [3]. I am intrigued to see how lessons learned by both researchers and publishers in this pandemic will affect the scholarly publishing landscape in the future.

Other content highlights from this year’s *Future Science OA* include a review of over 600,000 US patients with advanced or metastatic cancer, seeking to estimate the number who are eligible for/could respond to cytotoxic chemotherapy [4]; an analysis of opinions on elective egg freezing across the globe, which resulted in some interesting conclusions and recommendations [5]; and some interesting articles on data and standardization – the proposal of a new, open access natural products database [6] and an introduction to the PRE.M.I.S.E project, which is seeking to standardize data collection following brain radiation therapy [7].

This year saw us team up with our sister journal *BioTechniques* to make the Future Science Future Star Award – recognizing outstanding early career researchers – better than ever. This year saw Andy Tay Kah Ping, a bioengineer from the National University of Singapore, win and we are delighted to have him join us on our early career research advisory panel [8].

Finally, this year we also integrated with bioRxiv, meaning authors are now able to submit directly to the journal from that preprint server.

## Journal statistics

While we worked hard to prevent it, the COVID-19 pandemic did affect journal turnaround times this year, although I am pleased that it has been minimized by a commendable home-based effort from our team. On average, we have a desk decision to authors within 2 working days, accepted articles receive that decision 10 weeks after submission, and articles are published 7.5 weeks after acceptance. In 2020 we accepted 66.4% of submissions – down from 80% in 2019, although I note that COVID-19 submissions have been partially responsible for this dip.

As mentioned above, we have received over 1000 citations this year, up from the 787 we had received at the time of writing last year’s Foreword. This is an appreciable year-on-year increase.

**Figure 1.**
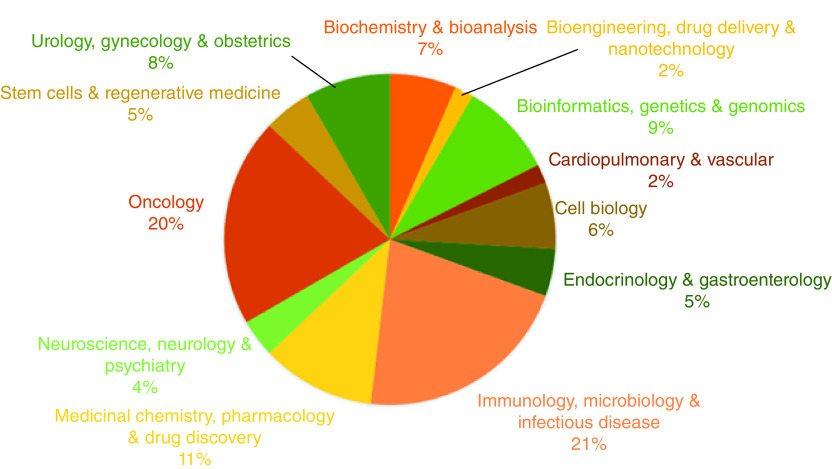
Topics covered in *Future Science OA* by percentage in 2020.

**Figure 2.**
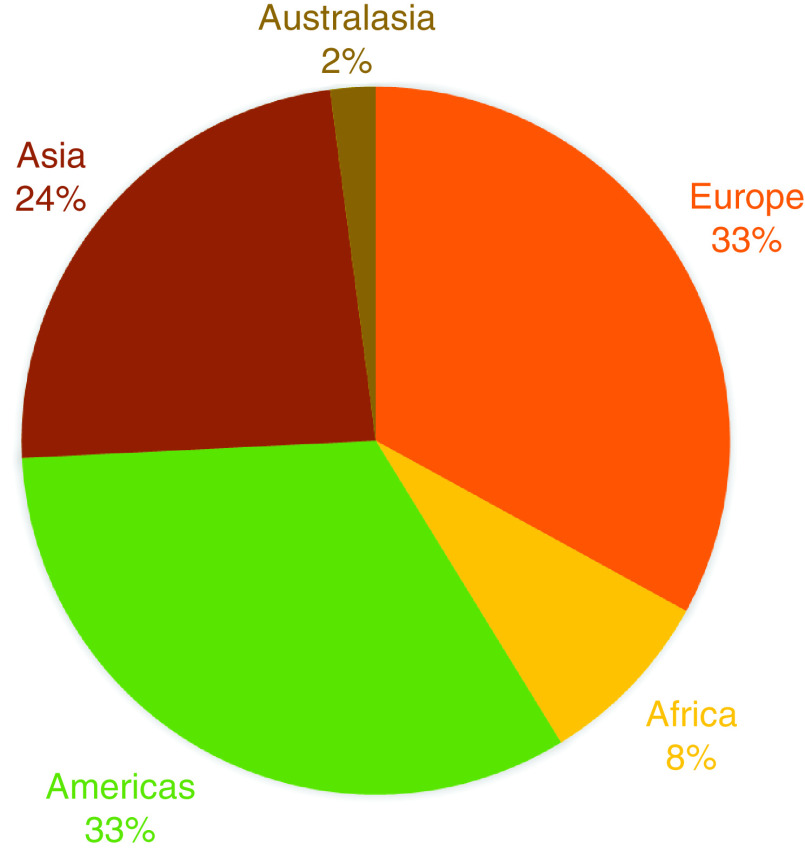
Geographic locations of *Future Science OA* corresponding authors in 2020.

**Figure 3.**
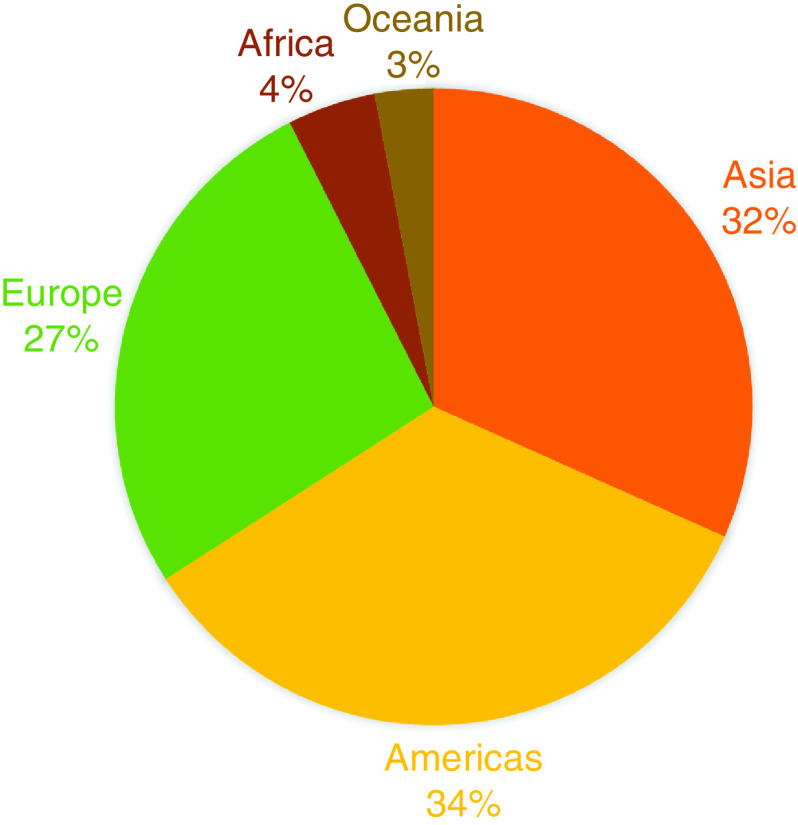
Location of readers of *Future Science OA*.

Topic areas covered in the journal continue to reflect the state of the biomedical field (Figure 1). Our author demographics remain consistent (Figure 2), and our readership remains global and, again, fairly consistent (Figure 3). These latter two demographics reflect our ability to provide authors from low-income countries with fee waivers, and the fact that we are open access and represented across most scholarly research search engines, ensuring access to as many readers as we can. We also continue to publish lay abstracts, which – we hope – goes some way to ensuring the public can understand the research their taxes may have helped produce.

## Thanks to our contributors

As always, we are hugely thankful to our contributors. In particular this year, a special mention goes to the over 350 researchers who have peer reviewed for us in 2020, despite being locked out of labs or working overtime due to COVID-19.

## Looking forward to 2021

My main hope for 2021 is that the COVID-19 pandemic is consigned to history – not necessarily a given, at this point. In better news, next year we look forward to more special focus issues, the next iteration of the Future Science Future Star Award, and working with more exciting researchers to perfect their publications. We are also currently working to make ourselves compliant with the requirements of Plan S. If you have any suggestions for topic coverage, special issues or collaborations in 2021, please get in touch.
